# Trends and Disparities in Acute Myocardial Infarction‐Related Mortality Among U.S. Adults With Hypertension, 2000–2023

**DOI:** 10.1002/clc.70129

**Published:** 2025-04-21

**Authors:** Maryam Sajid, Dua Ali, Shaheer Qureshi, Reja Ahmad, Asim Sajjad, Saad Ahmed Waqas, Raheel Ahmed, Peter Collins

**Affiliations:** ^1^ Department of Medicine Dow University of Health Sciences Karachi Pakistan; ^2^ Department of Medicine Ziauddin Medical University Karachi Pakistan; ^3^ National Heart and Lung Institute Imperial College London London UK

**Keywords:** acute myocardial infarction, cardiovascular diseases, CDC WONDER database, census region, gender, healthcare disparities, hypertension, mortality trends, race, urbanization

## Abstract

**Background:**

Hypertension is a major public health concern and a key risk factor for acute myocardial infarction (AMI), significantly contributing to cardiovascular mortality. Despite advancements in management and treatment, trends in associated mortality remain underexplored.

**Objective:**

This study examines U.S. national trends in hypertension‐ and AMI‐associated mortality from 2000 to 2023, focusing on demographics and regions.

**Methods:**

Age‐adjusted mortality rates (AAMRs) per 100,000 for adults aged ≥ 25 with hypertension and AMI were extracted from the CDC WONDER database. Annual percent changes (APCs) and average APCs (AAPCs) with 95% confidence intervals (CIs) were calculated, stratified by year, sex, race/ethnicity, age, urbanization, and Census region.

**Results:**

From 2000 to 2023, 933,024 hypertension‐ and AMI‐related deaths were recorded. Overall, AAMR declined from 19.84 per 100,000 in 2000 to 16.26 in 2023 (AAPC: −0.93%, 95% CI: −1.18% to −0.76%). However, a sharp rise in mortality occurred between 2018 and 2021, coinciding with the COVID‐19 pandemic. Stratified analyses revealed persistently higher mortality rates among menmen, non‐Hispanic BlackBlack individuals, and residents of the Southern and rural U.S. regions. Younger adults showed an increasing AAMR trend, indicating a growing burden of hypertension and AMI‐associated disease.

**Conclusion:**

While long‐term mortality trends show a decline, recent years have seen a rise, particularly among high‐risk groups. Targeted public health interventions addressing hypertension management, cardiovascular risk reduction, and healthcare disparities are essential to mitigate the ongoing burden of hypertension and AMI mortality in the U.S.

## Summary

- Hypertension and acute myocardial infarction (AMI)‐associated mortality trends show significant differences between men and women, with men experiencing higher rates.
- Black individuals have the highest mortality rates compared to other racial groups.
- Hypertension and AMI‐associated mortality trends vary across census regions, with the South showing steeper increases.
- Rural areas exhibit a sharper rise in mortality compared to urban areas.
- Older populations exhibit higher mortality rates compared to younger age groups.
- Findings highlight the need for targeted interventions in high‐risk groups.

## Graphical abstract

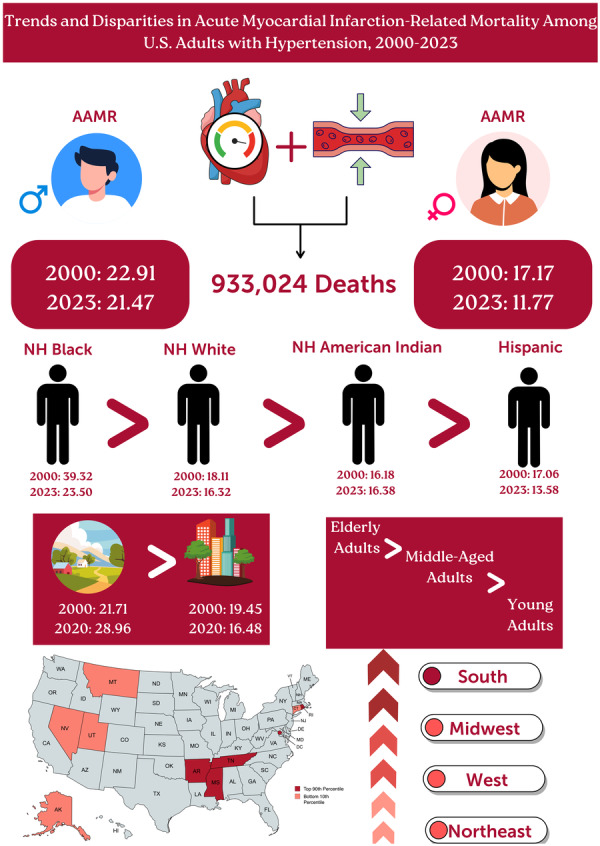

## 1 Introduction

Hypertension remains one of the most critical and pervasive risk factors for cardiovascular disease (CVD), affecting nearly half of all U.S. adults and contributing to substantial health and economic burdens [1]. Approximately 116 million (1 in 2) U.S. adults are affected by hypertension, yet only about one in four have it under control [2]. The treatment of hypertension imposes a significant financial burden, with annual costs for healthcare services, medications, and productivity losses due to premature death ranging from $131 billion to $198 billion [3]. It is predicted that by 2030, approximately 41.4% of adults in the United States will be affected by hypertension [4]. Similarly, Acute Myocardial Infarction (AMI) remains a major cause of mortality in developed countries. Globally, the condition affects nearly 3 million individuals, with the United States reporting over 1 million deaths each year [5].

The connection between hypertension and AMI presents a serious health concern. Numerous studies have consistently reported a significant rise in mortality associated with hypertension, with AMI being the primary cause [6, 7]. Studies have shown that approximately 50%–60% of patients hospitalized with AMI have a history of hypertension. High blood pressure, with robust evidence supporting its causal role in cardiovascular events, is one of the most well‐established risk factors for CVD. A history of hypertension is commonly observed in patients with AMI, often with a distinct risk profile compared to normotensive ischemic patients [8, 9].

The increasing prevalence of coexisting hypertension and AMI underscores the need to understand their interplay better to improve patient outcomes. However, trends in AMI‐associated mortality among patients with hypertension remain inadequately explored. This review aims to assess trends in hypertension‐ and AMI‐associated mortality in the United States from 2000 to 2023 and evaluate differences by sex, age, race, ethnicity, geographic region, and urbanization, using data from the Centers for Disease Control and Prevention's (CDC) Wide‐ranging Online Data for Epidemiologic Research (WONDER) [10].

### 1.1 Methodology

#### 1.1.1 Study Design and Database

We performed a retrospective cohort study aimed to assess the AMI‐associated mortality rate in patients with concomitant hypertension in the United States from 2000 to 2023. The Multiple Cause of Death database in CDC WONDER was analyzed to determine whether hypertension or AMI was listed as an underlying or contributing cause of death on nationwide death certificates [10]. Institutional review board approval was not required as CDC WONDER contains anonymized, publicly available data. We extracted hypertension‐ and AMI‐related death counts and population sizes from 2000 to 2023. Deaths were identified using ICD‐10 codes: I10‐I15 for hypertension and I21‐I22 for AMI. Additionally, we followed the guidelines established by the reporting standards of the Strengthening the Reporting of Observational Studies in Epidemiology (STROBE) [11].

#### 1.1.2 Demographic and Geographical Study Groups

Specifically, data extracted for analysis included gender, race, age groups, urbanization level, state, and census region. Genders included males and females. Race groups were divided into NH White, NH Black or African American, NH American Indian or Alaska Native, and Hispanic or Latino [12, 13]. Age groups were categorized as adults aged (25–44) years (younger adults), 45–64 years (middle‐aged adults), and 65+ (older adults). The population was divided into urban (large metropolitan area, medium/small metropolitan area) and rural (population < 50,000) counties according to the 2013 U.S. census classification. Regions were classified as Northeast, Midwest, South, and West based on U.S. Census Bureau definitions [14]. For the primary cause of death, we used the rankable cause of death feature in the CDC WONDER database, which provides mortality counts and rates for the 15 leading causes of death among individuals with these conditions.

### 1.2 Statistical Analysis

Crude mortality rates (CMR) and age‐adjusted mortality rates (AAMR) for hypertension and AMI‐associated deaths were calculated. The CMR was determined by dividing the number of deaths by the corresponding U.S. population, while the AAMR accounted for variations in age distribution, facilitating a standardized comparison. The United States population as of the year 2000 was used as the standard population for AAMR calculations [15]. The Joinpoint Regression Program (Joinpoint version 5.2.0) was used to assess trends in Hypertension and AMI‐associated mortality over the study period [15]. This program identifies significant changes in annual mortality trends over time through Joinpoint regression, fitting linear segments where notable temporal variations occurred. Annual percentage change (APC) and 95% confidence intervals (CIs) for AAMRs were calculated using the Monte Carlo permutation test. The weighted average of the APCs was summarized as the average annual percent change (AAPC), with corresponding 95% CIs to capture the overall mortality trend for the 2000–2023 study period. A two‐tailed *t*‐test was conducted to evaluate whether APC and AAPCs indicated an increase or decrease in mortality during the study period. Statistical significance was set at *p* ≤ 0.05 and is reported in the results, figures, and Supporting Information. We also performed sensitivity analyses by considering AMI alone as the contributing or underlying cause of death, with hypertension as the multiple cause of death. The study did not require approval from the local institutional review board because it used an anonymous public data set provided by the government.

## 2 Results

From 2000 to 2023, a total of 933,024 deaths were recorded due to hypertension and AMI (Table 1). Of these, 507,105 (54.35%) occurred in men and 425,919 (45.65%) in women. By race/ethnicity, 140,894 deaths were among NH Blacks, 696,539 among NH Whites, 5,191 among NH American Indian or Alaska Natives, and 62,570 among Hispanics. Age distribution showed 17,296 (1.85%) deaths in young adults (25–44), 209,315 (22.43%) in middle‐aged adults (45–64), and 706,413 (75.71%) in older adults (65+ ) (Supporting Information S1: Table S1). Location of death was recorded for 895,358 cases, with 49.56% occurring in medical facilities, 12.85% in nursing homes/long‐term care facilities, 1.24% in hospices, and 35.35% at decendent's home (Supporting Information S1: Tables S2, S6).

**Table 1:** Frequency and age adjusted rates per 100,000 in adults with hypertension and AMI concomitantly stratified by sex, race, census region and location of death.

	Deaths	Population	AAMR 2000 (95% CI)	AAMR 2023 (95% CI)	AAPC (95% CI)
Overall	933,024	4.983E + 09	19.84 (19.63–20.04)	16.26 (16.11–16.24)	−0.93 (−1.18 to −0.76)
Sex					
Male	507,105	2.405E + 09	22.91 (22.56–23.27)	21.47 (21.21–21.73)	−0.35 (−0.59 to −0.16)
Female	425,919	2.578E + 09	17.17 (16.92–17.42)	11.77 (11.60–11.94)	−1.71 (−1.98 to −1.54)
Non‐Hispanic race					
NH White	696,539	3.105E + 09	18.11 (17.90–18.33)	16.32 (16.14–16.50)	−0.48 (−0.71 to −0.31)
NH Black/African American	140,894	582396578	39.32 (38.3–40.33)	23.50 (22.91–24.09)	−2.36 (−2.74 to −2.08)
NH American Indian or Alaska Native	5,191	36666308	16.81 (13.19–19.17)	16.38 (14.43–18.33)	0.13 (0.44 to 0.82)
Hispanic Race	62,570	684525097	17.06 (16.17–17.96)	13.58 (13.14–14.03)	−1.33 (−1.82 to −0.82)
Census Region					
Northeast	155,162	912592124	18.44 (18.01–18.87)	11.92 (11.61–12.22)	−2.01 (−2.37 to −1.73)
Midwest	209,198	1.07E + 09	21.09 (20.66–21.53)	15.37 (15.04–15.69)	−1.28 (−1.55 to −1.10)
South	381,979	1.852E + 09	20.96 (20.60–21.31)	19.97 (19.70–20.25)	−0.17 (−0.42 to 0.09)
West	186,685	1.148E + 09	17.87 (17.43–18.31)	14.29 (13.99–14.59)	−1.12 (−1.61 to −0.82)
Location					
Medical facility	443,762	—	—	—	—
Hospice facility	11,064	—	—	—	—
Nursing home/long term care	115,063	—	—	—	—
Decedent's home	325,469	—	—	—	—

*Note:* Represents data unavailable from CDC WONDER.

### 2.1 Annual Trends for Hypertension and AMI‐Associated AAMR

**Figure 1:**
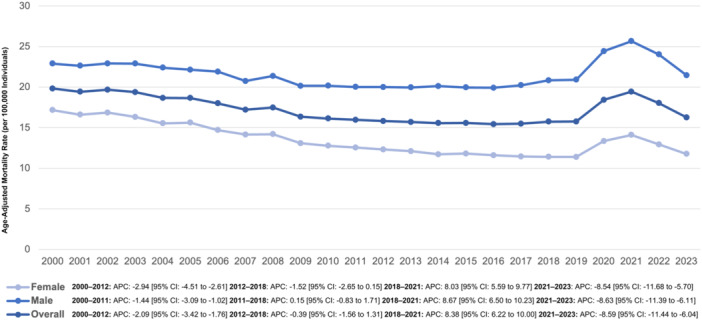
Hypertension and AMI‐associated AAMRs per 100,000 stratified by sex in the United States from 2000 to 2023.

Overall, there was a modest decline in AAMR due to hypertension and AMI, with the AAMR decreasing from 19.84 in 2000 to 16.26 in 2023 (AAPC −0.93% [95% CI: −1.18% to −0.76%]). Initially, AAMR saw a pronounced decline from 2000 to 2012 (APC −2.09% [95% CI: −3.42% to −1.76%]), followed by a period of stability until 2018 (APC −0.39% [95% CI: −1.59% to 1.31%]). However, a substantial surge occurred between 2018 and 2021 (APC 8.38% [95% CI: 6.22%–10.00%]), followed by a significant downturn from 2021 to 2023 (APC −8.59% [95% CI: −11.44% to −6.04%]) (Figure 1, Supporting Information S1: Tables S4, S11). Upon sensitivity analysis, AMI as an underlying or contributing cause of death with hypertension as the multiple cause of death showed a similar trend. This suggests that the observed decrease in mortality was not driven by a general decline in AMI‐associated deaths but may instead be influenced by hypertension.

### 2.2 Hypertension and AMI‐Associated AAMR Stratified by Sex

Among males, AAMR declined from 22.91 in 2000 to 20.02 in 2011 (APC −1.44% [95% CI: −3.09% to −1.02%]), followed by a slight uptick to 20.85 in 2018 (APC 0.15% [95% CI: −0.83% to 1.71%]). A sharp escalation occurred as AAMR peaked at 25.67 in 2021 (APC 8.67% [95% CI: 6.50%–10.23%]), before experiencing a marked decline to 21.47 in 2023 (APC −8.63% [95% CI: −11.39% to −6.11%]).

Among females, AAMR initially showed a steady reduction from 17.17 in 2000 to 12.31 in 2012 (APC −2.95% [95% CI: −4.51% to −2.61%]). This downward trend continued nonsignificantly, reaching 11.41 in 2018 (APC −1.52% [95% CI: −2.65% to 0.15%]), before a significant increase to 14.11 in 2021 (APC 8.03% [95% CI: 5.59%–9.77%]). A sharp decline followed, bringing the AAMR down to 11.77 in 2023 (APC −8.54% [95% CI: −11.68% to −5.70%]) (Figure 1, Supporting Information S1: Tables S4, S11).

### 2.3 Hypertension and AMI‐Associated AAMR Stratified by Race

Among NH Whites, AAMR declined steadily from 18.11 in 2000 to 15.14 in 2012 (APC −1.68% [95% CI: −2.78% to −1.36%]). It then stabilized, hovering around 15.49 in 2018 (APC 0.03% [95% CI: −1.16% to 1.64%]), before a sharp spike to 19.09 in 2021 (APC 8.10% [95% CI: 5.98%–9.63%]). This surge was followed by a notable decline, bringing AAMR down to 16.32 in 2023 (APC −6.85% [95% CI: −9.61% to −4.35%]).

Among Hispanics or Latinos, AAMR showed a consistent downward trajectory from 17.06 in 2000 to 13.01 in 2018 (APC −1.88% [95% CI: −2.50% to −1.39%]). However, this long‐term decline was abruptly interrupted by a sharp rise to 17.31 in 2021 (APC 12.39% [95% CI: 7.24%–16.02%]), followed by a significant drop to 13.58 in 2023 (APC −14.60% [95% CI: −20.06% to −8.34%]).

Among NH Black or African Americans, AAMR decreased substantially from 39.32 in 2000 to 25.30 in 2012 (APC −3.85% [95% CI: −6.21% to −3.31%]), with the decline continuing nonsignificantly to 23.47 in 2018 (APC −1.61% [95% CI: −3.48% to 0.83%]). This trend then reversed, with a steep increase to 29.38 in 2021 (APC 9.33% [95% CI: 5.78%–11.93%]), before AAMR declined sharply again to 23.50 in 2023 (APC −11.70% [95% CI: −15.95% to −7.58%]).

**Figure 2:**
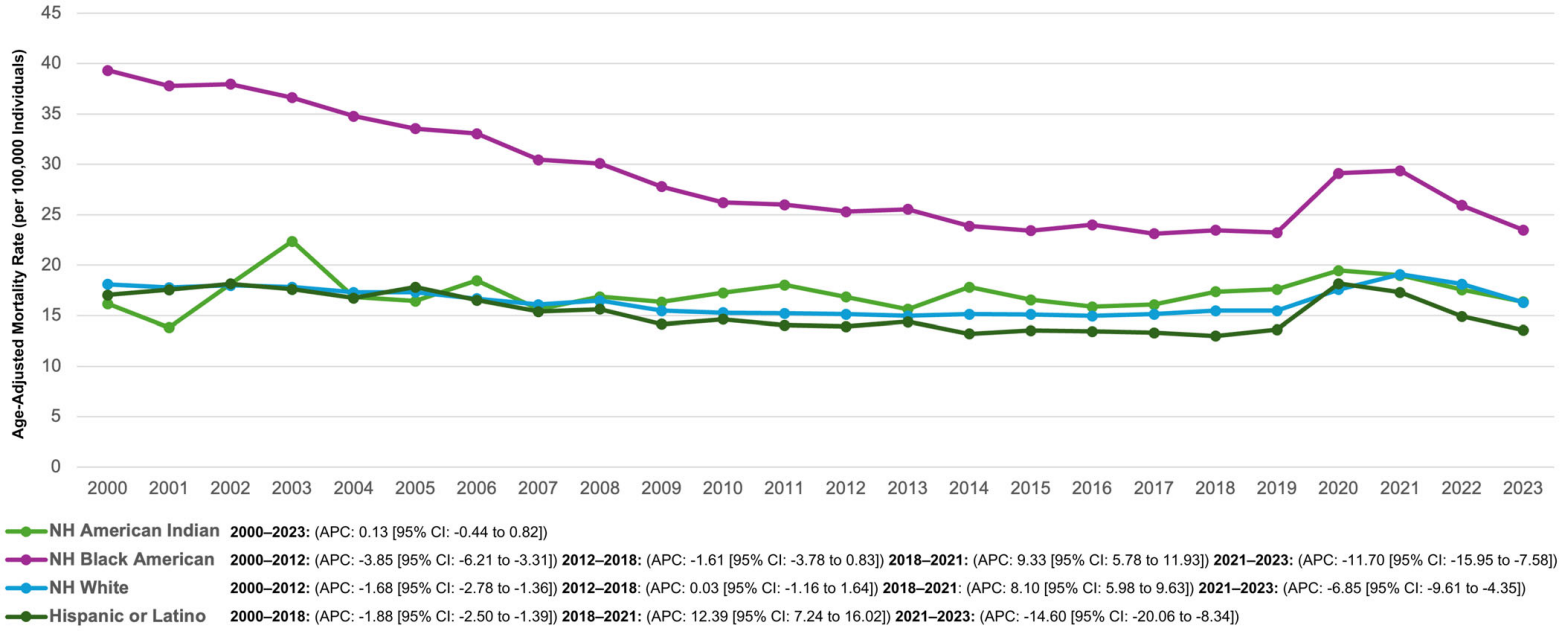
Hypertension and AMI‐associated AAMRs per 100,000 stratified by race in the United States from 2000 to 2023.

Among NH American Indians, AAMR exhibited a gradual but consistent increase in AAMR from 16.18 in 2000 to 16.38 in 2023 (APC 0.13% [95% CI: −0.44% to 0.82%]) (Figure 2, Supporting Information S1: Tables S5, S11).

#### 2.3.1 Hypertension and AMI‐Associated AAMR Stratified by Census Region

Adults in different census regions consistently showed varying age‐adjusted mortality rates related to AMI‐induced hypertension throughout the study period. In the Northeast, the AAMR decreased from 18.44 (95% CI: 18.01–18.87) in 2000 to 11.92 (95% CI: 11.61–12.22) in 2023, with an AAPC of −2.01% (95% CI: −2.37 to −1.73). Similarly, in the Midwest, the AAMR declined from 21.09 (95% CI: 20.66–21.53) in 2000 to 15.37 (95% CI: 15.04 to 15.69) in 2023, with an AAPC of −1.28% (95% CI: −1.55 to −1.10). In the South, the AAMR decreased from 20.96 (95% CI: 20.60–21.31) in 2000 to 19.97 (95% CI: 19.70–20.25) in 2023, with an AAPC of −0.17% (95% CI: −0.42 to 0.09). The West also saw a decline in AAMR from 17.87 (95% CI: 17.43–18.31) in 2000 to 14.29 (95% CI: 13.99–14.59) in 2023, with an AAPC of −1.12% (95% CI: −1.61 to −0.82) (Supporting Information S1: Figure 2, Supporting Information S1: Tables S9, S11).

#### 2.3.2 Hypertension and AMI‐Associated AAMR Stratified by State

**Figure 3:**
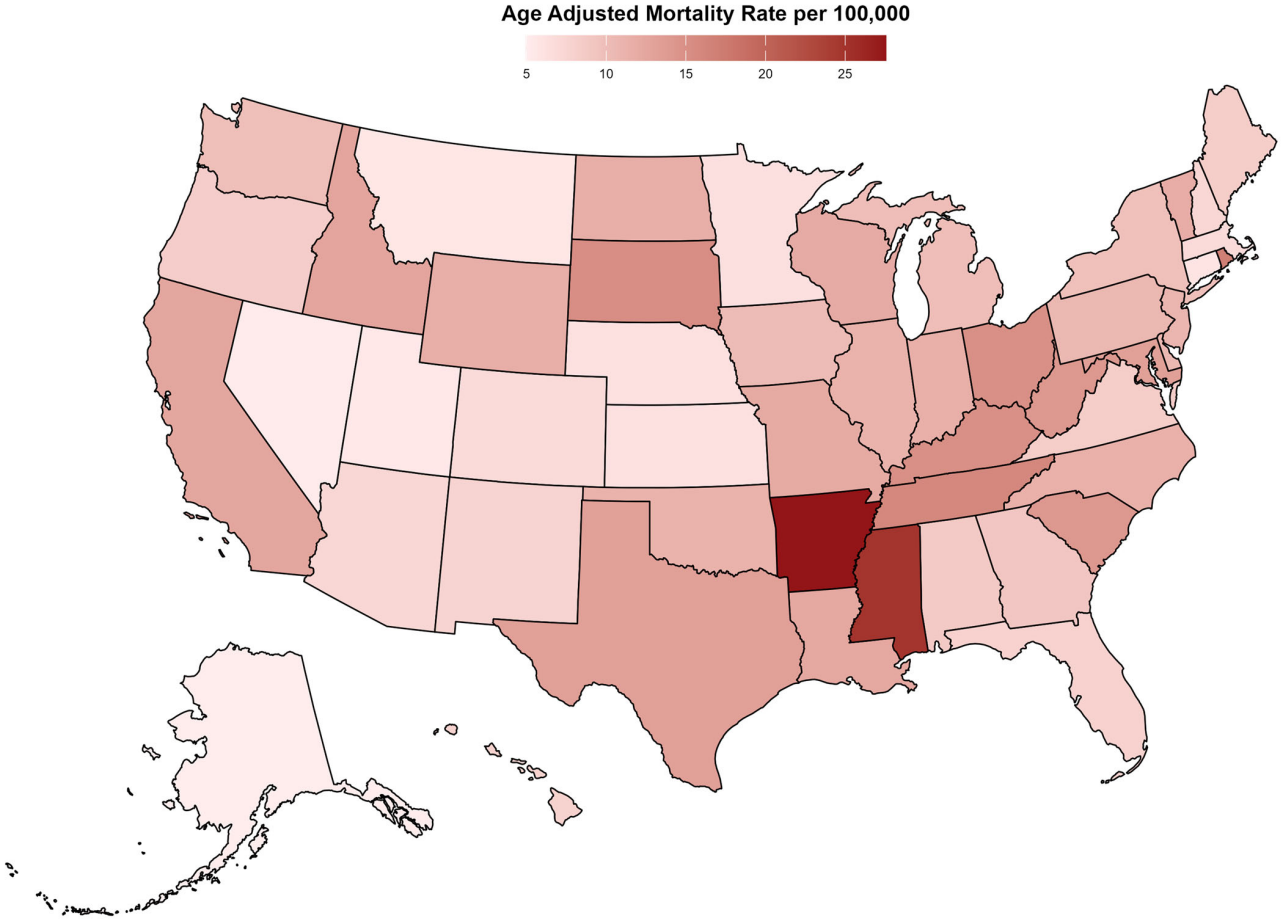
Hypertension and AMI‐associated AAMRs per 100,000 stratified by State in the United States from 2000 to 2019.

AAMR varied widely across states from 2000 to 2019, ranging from a low of 4.97 in Alaska (95% CI: 4.49–5.46) to a high of 27.55 in Arkansas (95% CI: 27.14–27.95). States in the top 90th percentile included Arkansas (27.55), Mississippi (24.86), Rhode Island (16.81), Tennessee (15.89), and the District of Columbia (15.52), with rates nearly three to five times higher than states in the 10th percentile—Alaska (4.97), Nevada (5.18), Utah (5.50), Montana (5.65), and Connecticut (5.81) (Figure 3, Supporting Information S1: Table S8).

#### 2.3.3 Hypertension and AMI‐Associated AAMR Stratified by Urbanization

In metropolitan areas, the AAMR declined from 19.45 in 2000 to 14.91 in 2011 (APC: −2.59% [95% CI: −3.98% to −2.18%]), followed by a slower decline to 14.31 in 2018 (APC: −0.95% [95% CI: −2.00% to 0.62%]). However, this trend reversed with an increase to 16.48 in 2020 (APC: 7.53% [95% CI: 3.20%–10.05%]).

In nonmetropolitan areas, AAMR remained relatively stable from 21.71 in 2000 to 20.96 in 2013 (APC: −0.38% [95% CI: −2.32% to 1.69%]), followed by a slight increase to 23.46 in 2018 (APC: 1.58% [95% CI: −0.43% to 3.42%]). The rise accelerated significantly from 2018 to 2020, reaching 28.96 (APC: 10.81% [95% CI: 5.58%–14.10%]) (Supporting Information S1: Figure S3, Supporting Information S1: Tables S7, S11).

#### 2.3.4 Hypertension and AMI‐Associated AAMR Stratified by Age

Among younger adults, AAMR showed a modest increase from 0.75 in 2000 to 0.96 in 2018 (APC: 1.11% [95% CI: 0.50%–1.54%]), followed by a peak at 1.28 in 2021 (APC: 11.48% [95% CI: 6.82%–14.26%]) before declining to 1.02 in 2023 (APC: −10.91% [95% CI: −17.55% to −3.56%]).

Among middle‐aged adults, AAMR initially declined from 10.39 in 2000 to 9.53 in 2010 (APC: −0.79% [95% CI: −3.69% to −0.001%]) before experiencing a nonsignificant increase to 10.61 in 2018 (APC: 0.97% [95% CI: −0.30% to 2.93%]). It then surged to 13.15 in 2021 (APC: 8.56% [95% CI: 5.64%–10.52%]) before dropping to 10.88 in 2023 (APC: −9.09% [95% CI: −13.21% to −5.08%]).

Among older adults, AAMR declined from 81.46 in 2000 to 79.24 in 2003 (APC: −0.76% [95% CI: −2.19% to 2.17%]) before decreasing further to 62.54 in 2011 (APC: −3.01% [95% CI: −4.90% to −2.54%]). After another decline to 59.66 in 2018 (APC: −0.97% [95% CI: −1.74% to 0.33%]), AAMR rose to 73.41 in 2021 (APC: 8.23% [95% CI: 6.50%–9.49%]) before dropping again to 61.69 in 2023 (APC: −8.27% [95% CI: −10.58% to −6.28%]) (Supporting Information S1: Figure S1, Supporting Information S1: Tables S3, S11).

#### 2.3.5 Top Underlying Causes of Death

We assessed the top 15 underlying causes of death in patients with hypertension and AMI. Among these, diseases of the heart were the leading underlying cause of death (705,869 deaths), followed by diabetes mellitus (94,958 deaths), and chronic lower respiratory diseases (14,172) deaths (Supporting Information S1: Table S10).

## 3 Discussion

The present study on mortality trends among individuals with hypertension and AMI from 2000 to 2023 reveals several significant findings (Central Illustration 1). These results highlight the intricate relationship between cardiovascular outcomes and their subtypes, underscoring critical areas for targeted interventions. Over the past two decades, mortality related to hypertension and AMI has declined; however, the reduction was less pronounced among males compared to the overall population. Among racial groups, NH Black individuals exhibited the highest mortality rates, while NH White individuals experienced the least improvement. Geographically, the Southern region and rural areas recorded higher mortality rates than other regions.

**Central Illustration 1:**
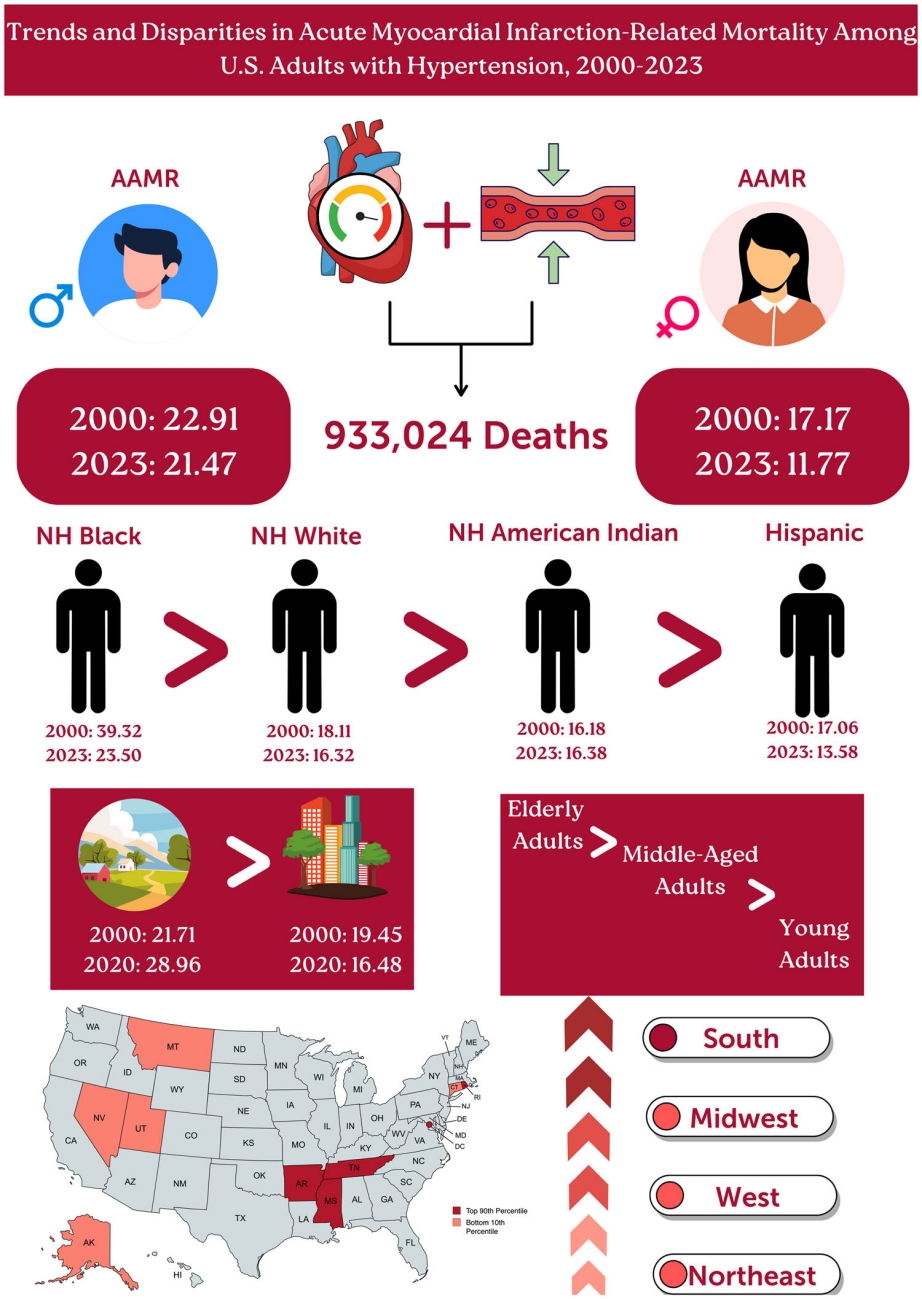
Trends and disparities in acute myocardial infarction‐related mortality among U.S. adults with hypertension, 2000–2023.

The trend in AAMR for hypertension AMI followed an irregular pattern. Between 2000 and 2012, AAMR steadily declined, followed by a plateau phase from 2014 to 2018. However, after 2019, there was a sharp increase in AAMR until 2021, followed by a rapid decline through 2023. These findings align with the findings of Abrams et al. who reported a stagnation in cardiovascular mortality during the post‐2010 period [16]. A major contributor to this stagnation may be the increasing hypertension‐related mortality observed over the past two decades [17]. This may also be explained by the rising prevalence of lifestyle‐related and cardiometabolic conditions, such as obesity and diabetes, which are major underlying factors in both hypertension and AMI [18, 19, 20]. Another potential component is the increased life expectancy of people with hypertension due to advancements in cardiovascular treatment in recent years, hence prolonged exposure to hypertension leading to more cases of AMI [21].

The spark rise in AAMR across all demographics in 2019 can be attributed to the COVID‐19 pandemic. Individuals with preexisting health conditions, particularly those with hypertension and AMI, were disproportionately affected, facing a significantly higher risk of severe illness and mortality. Beyond placing immense strain on healthcare systems, the pandemic exacerbated existing health disparities, potentially leading to delays or inadequacies in the management of chronic diseases [22]. Populations already experiencing high rates of hypertension and AMI were particularly impacted [23]. Hispanic or Latino individuals, older adults, and rural residents experienced the most pronounced increases in mortality, reflecting how healthcare disparities and vulnerabilities within these communities were magnified, ultimately leading to significant loss of life. The pandemic disproportionately affected those already at heightened risk, exposing deep‐rooted inequities within the healthcare system.

Hypertension significantly contributes to AMI through multiple interrelated mechanisms. Chronic hypertension induces endothelial dysfunction, fostering a pro‐inflammatory environment that accelerates atherosclerosis, increasing the risk of plaque rupture and thrombus formation, which can acutely occlude coronary blood flow [24, 25]. Additionally, prolonged pressure overload leads to left ventricular hypertrophy, increasing myocardial oxygen demand while compromising coronary perfusion, making the heart more susceptible to ischemic injury [26, 27, 28]. Furthermore, autonomic nervous system dysregulation associated with hypertension contributes to increased sympathetic activity, resulting in elevated heart rate and peripheral vasoconstriction, further exacerbating myocardial stress [29]. Moreover, hypertension impairs microvascular function, reducing myocardial perfusion and increasing ischemic vulnerability, even without significant epicardial coronary artery disease [30].

Our analysis identified that the AAMR associated with hypertension and AMI was marginally higher in men than in women. This finding contrasts with previous literature, which suggests that women with hypertension and AMI often experience worse in‐hospital and long‐term prognoses than men [31, 32, 33, 34]. The higher mortality observed in men may be primarily attributed to their greater incidence of hypertension [17]. Additionally, studies indicate that estrogen provides cardioprotective effects in women, which may explain why men are more prone to develop heart disease earlier in life, compared to women [35]. Furthermore, other factors such as poorer glycemic control due to higher diabetes prevalence, as well as unhealthy behaviors like smoking and excessive alcohol consumption—often exacerbated by social and competitive pressures—may contribute to the increased risk of cardiovascular mortality in men [19, 36].

Our data also highlight significant racial and ethnic disparities among patients. NH Black individuals had the highest AAMRs throughout the study period. The persistently elevated AAMRs in Black populations may be attributed to a greater prevalence of adverse health behaviors, including poor diet quality, inadequate adherence to recommended physical activity, untreated sleep disorders leading to insufficient sleep, and higher rates of obesity and diabetes [37, 38, 39, 40]. These risk factors often cluster within Black or African American adults, compounded by social and environmental determinants of health and neighborhood characteristics that further contribute to higher mortality [41, 42]. Additionally, Candelaria et al. reported that ethnic minorities are half as likely to complete cardiac rehabilitation, making them more vulnerable to severe complications from hypertension and AMI [43]. Interestingly, White patients not only experienced the smallest decline in mortality during the study period but also had the slowest recovery from COVID‐19 compared to other racial groups. The opioid epidemic and rising rates of metabolic syndrome—particularly among middle‐aged and rural White populations—may have offset improvements in cardiovascular mortality [44, 45]. Moreover, the increasing prevalence of “deaths of despair”—including suicide, drug overdoses, and alcohol‐related liver disease—among White individuals may have further influenced mortality trends [46].

Contrary to all demographic groups, we observed a concerning increase in AAMRs among younger adults during the study period. Evidence suggests that poor diet, unhealthy lifestyle choices, and chronic stress are critical contributors to the early onset of hypertension and AMI [47, 48]. Additionally, the ongoing obesity and diabetes epidemic among this age group further exacerbates cardiovascular conditions [49]. Despite continuous advancements in the diagnosis and treatment of hypertension and AMI over the past two decades, the rising mortality rates among patients > 65 years remain a major concern. Furthermore, the COVID‐19 pandemic contributed to a significant excess in hypertension‐ and AMI‐related deaths among older adults, further compounding the already high mortality rates in this population [50].

We observed significant regional variations in AAMRs, with the highest rates reported in the South, followed by the Midwest, West, and Northeast. Notably, Arkansas and Mississippi exhibited particularly high AAMRs. Also, mortality rates were substantially higher in rural areas compared to urban regions. These disparities are largely driven by regional healthcare inequalities, socioeconomic factors, and lifestyle differences [51, 52, 53]. Limited access to preventive care in rural communities contributes to elevated mortality rates despite advancements in cardiovascular healthcare [54]. Additionally, lower healthcare capacity in these regions results in inadequate cardiovascular care, while socioeconomic status further influences the prevalence and severity of cardiovascular disease [55]. The impact of COVID‐19 further exacerbated these disparities, with rural areas witnessing a doubling of the AAMR increase compared to urban centers.

Future public health initiatives can be guided by the trends in age‐adjusted hypertension‐ and AMI‐related mortality identified in this study. The initial sharp decline in mortality may be attributed to reduced smoking rates, advancements in medical and surgical treatment, and aggressive cholesterol management through awareness programs such as the *Million Hearts* initiative, which enhanced cardiovascular disease prevention efforts across both the public and private sectors [56, 57]. However, the recent rise in AAMR is concerning. In response, several guidelines have been developed to enhance the identification and management of patients with hypertension and AMI. The 2023 American College of Cardiology (ACC)/American Heart Association (AHA) blood pressure guidelines recommend maintaining a target blood pressure below 130/80 mmHg through lifestyle modifications such as a healthy diet, regular physical activity, and weight management [58]. Moreover ACC and AHA guidelines suggest calcium channel blockers and thiazide diuretics as first‐line therapy for non‐Hispanic BlackBlack adults with hypertension who do not have heart failure or renal disease, with an added emphasis on the use of antihypertensive medications. This aims to counter the increased mortality in this racial group [59]. Other essential strategies include increasing statin use for hypertension control, which has risen by 149%, contributing positively to hypertension management [60]. Additionally, improving AMI outcomes through early mechanical reperfusion with percutaneous coronary intervention or pharmacological reperfusion, along with adjunctive therapy using antiplatelets and antithrombotics, can enhance post‐AMI survival [61]. Further policy measures should focus on making cardiovascular treatment more affordable through expanded insurance coverage and the regulation of dietary risk factors, such as reducing trans‐fat and sodium content in processed foods. Implementing these strategies effectively can significantly enhance hypertension and AMI care in the United States.

## 4 Limitations

Our study had certain limitations; firstly, the data retrieved relied solely on ICD‐10 codes assigned by WHO which may be prone to omissions or misrepresentations. Certain variables such as the socioeconomic status of patients were not assessed due to the absence of said data despite it being one of the critical determinants while assessing healthcare. The database did not contain laboratory and clinical findings or the treatment history of patients which would have enabled us to provide a further comprehensive overview. Lastly, the urbanization data were not available after 2020. Similarly, state‐level data were only collected up to 2019 to prevent the impact of COVID‐19 deaths.

## 5 Conclusion

Our study reveals that following an initial steep decline, there has been a recent increase in hypertension‐ and AMI‐related mortality in the United States, particularly during the COVID‐19 pandemic. The highest risk of mortality is observed among men and NH Black/AA individuals. Additionally, rural areas and the Southern region of the U.S. report the highest AAMR compared to other regions. These findings underscore the interconnected nature of cardiovascular conditions and social determinants of health. To improve outcomes across all population groups, healthcare strategies should be designed to promote health equity, particularly during public health crises such as the COVID‐19 pandemic Central Illustration 1.

## Ethics Statement

The authors have nothing to report.

## Conflicts of Interest

The authors declare no conflicts of interest.

## Supporting information

supplementary content htn ami revised.

## Data Availability

The data that support the findings of this study are openly available in CDC WONDER at https://wonder.cdc.gov/.
