# Supplementary material for: Trends and Disparities in Acute Myocardial Infarction‐Related Mortality Among U.S. Adults With Hypertension, 2000–2023
Source: Clin Cardiol. 2025 Apr 21;48(4):e70129. doi: 10.1002/clc.70129 (PMC12012249; doi:10.1002/clc.70129)
Supplement: Supplementary file 1 — supplementary content htn ami revised. [file CLC-48-e70129-s001.docx]

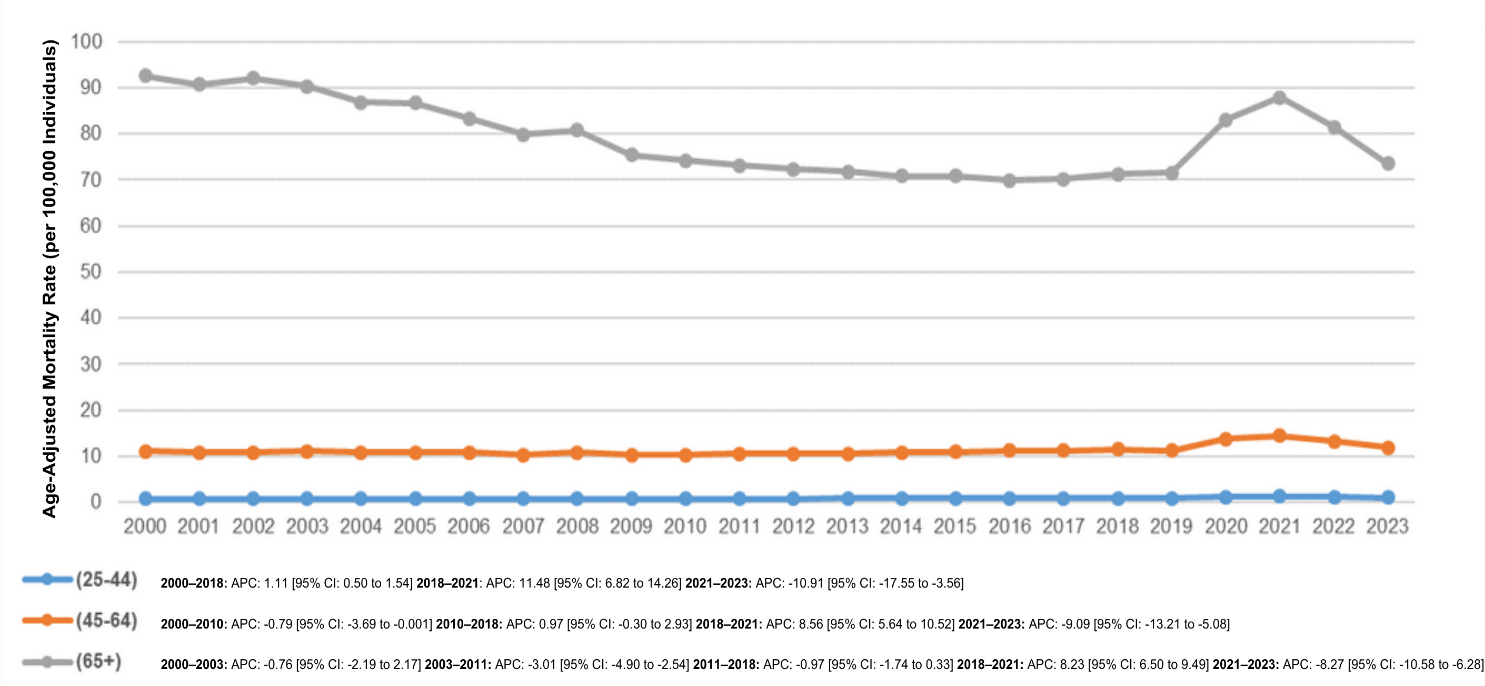


**Supplementary Figure 1.** Hypertension and AMI-associated AAMRs per 100,000 stratified by age in the United States from 2000 to 2023.


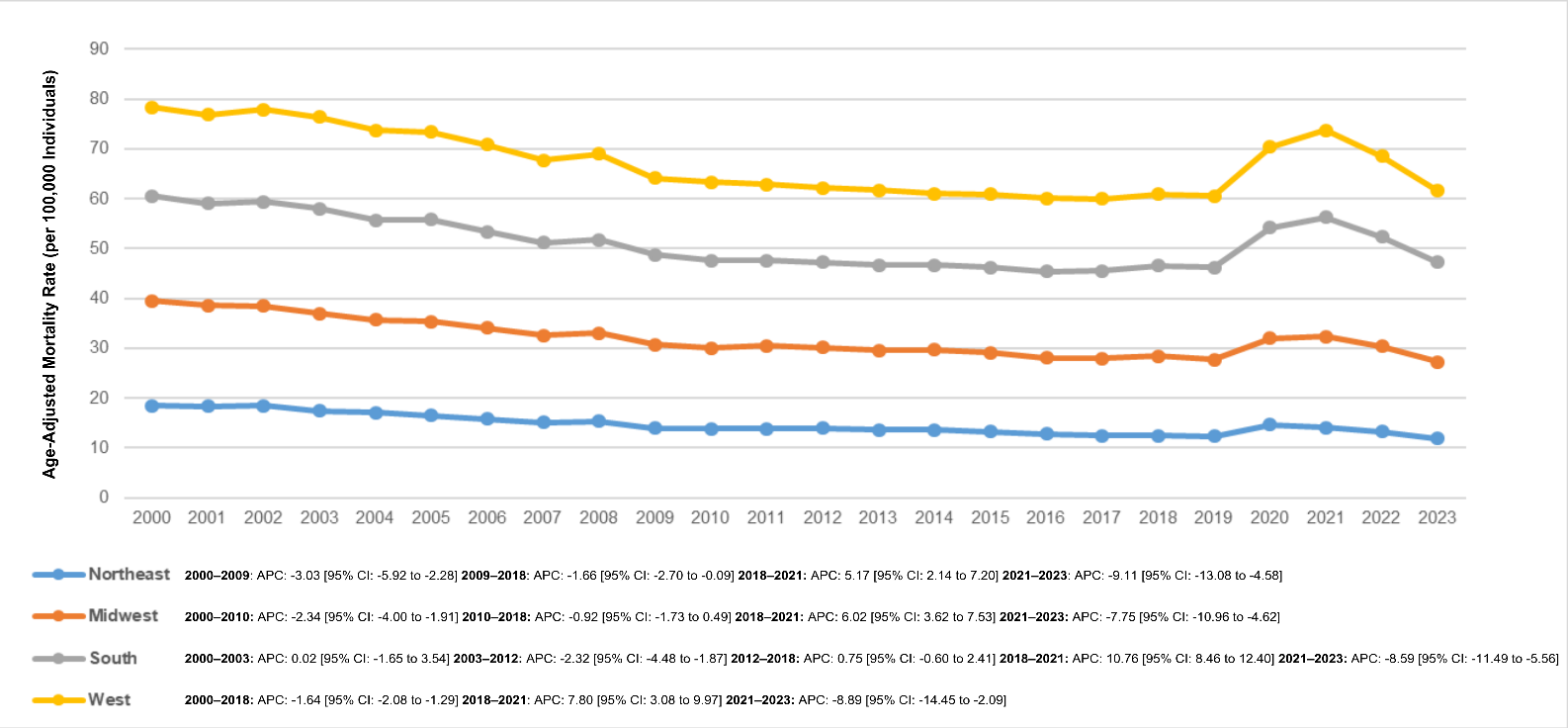


**Supplementary Figure 2.** Hypertension and AMI-associated AAMRs per 100,000 stratified by census region in the United States from 2000 to 2023.


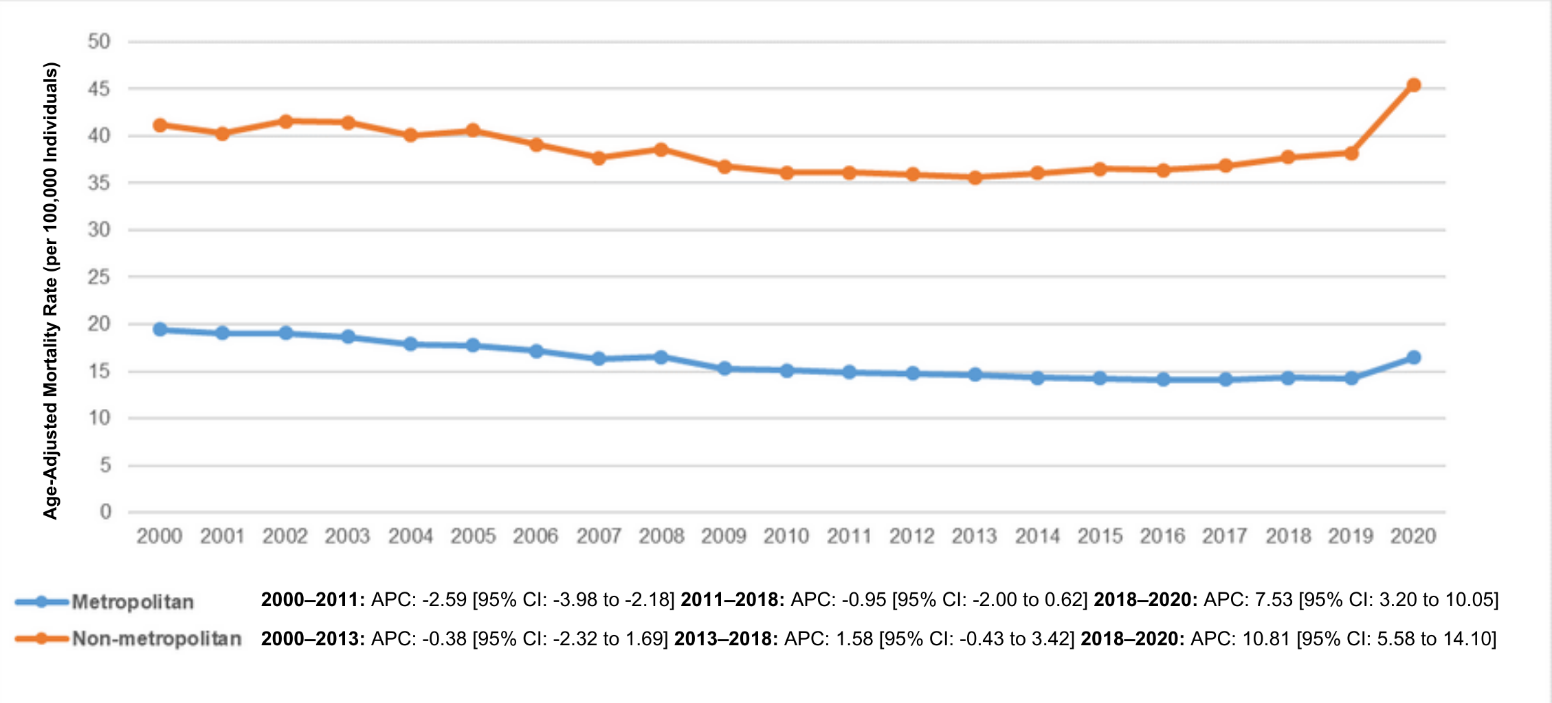


**Supplementary Figure 3.** Hypertension and AMI-associated AAMRs per 100,000 stratified by urbanization in the United States from 2000 to 2020.

|  | **Overall** | **Female** | **Male** | **Hispanic** | **NH American Indian** | **NH Black** | **NH White** | **Population** |
| --- | --- | --- | --- | --- | --- | --- | --- | --- |
| **2000** | 35455 | 18766 | 16689 | 1506 | 124 | 5916 | 27144 | 181984640 |
| **2001** | 35296 | 18462 | 16834 | 1624 | 113 | 5822 | 26946 | 184305128 |
| **2002** | 36319 | 18969 | 17350 | 1785 | 151 | 5928 | 27653 | 186208028 |
| **2003** | 36465 | 18635 | 17830 | 1814 | 188 | 5872 | 27745 | 188090429 |
| **2004** | 35699 | 17902 | 17797 | 1803 | 149 | 5699 | 27221 | 190205384 |
| **2005** | 36431 | 18372 | 18059 | 2024 | 157 | 5645 | 27690 | 192551384 |
| **2006** | 35850 | 17526 | 18324 | 1992 | 184 | 5725 | 27082 | 195019359 |
| **2007** | 35032 | 17245 | 17787 | 1933 | 155 | 5389 | 26613 | 197403777 |
| **2008** | 36416 | 17585 | 18831 | 2083 | 175 | 5501 | 27693 | 199795090 |
| **2009** | 34756 | 16507 | 18249 | 2007 | 171 | 5226 | 26411 | 202107016 |
| **2010** | 34901 | 16301 | 18600 | 2164 | 191 | 5060 | 26447 | 203891983 |
| **2011** | 35563 | 16399 | 19164 | 2243 | 221 | 5227 | 26847 | 206592936 |
| **2012** | 36095 | 16392 | 19703 | 2354 | 214 | 5252 | 27196 | 208826037 |
| **2013** | 36631 | 16452 | 20179 | 2581 | 207 | 5461 | 27286 | 211085314 |
| **2014** | 37200 | 16242 | 20958 | 2532 | 241 | 5328 | 27943 | 213809280 |
| **2015** | 38091 | 16700 | 21391 | 2758 | 252 | 5418 | 28457 | 216553817 |
| **2016** | 38558 | 16661 | 21897 | 2886 | 244 | 5729 | 28537 | 218641417 |
| **2017** | 39527 | 16784 | 22743 | 3043 | 243 | 5715 | 29185 | 221447331 |
| **2018** | 41018 | 17009 | 24009 | 3082 | 290 | 5943 | 30304 | 223311190 |
| **2019** | 41878 | 17300 | 24578 | 3332 | 286 | 6051 | 30847 | 224981167 |
| **2020** | 49722 | 20414 | 29308 | 4641 | 337 | 7779 | 35221 | 226635013 |
| **2021** | 51464 | 20821 | 30643 | 4508 | 322 | 7726 | 36903 | 228238412 |
| **2022** | 49607 | 20192 | 29415 | 4066 | 292 | 7002 | 36428 | 229508599 |
| **2023** | 45050 | 18283 | 26767 | 3809 | 284 | 6480 | 32740 | 231529762 |
| **Total** | 933024 | 425919 | 507105 | 62570 | 5191 | 140894 | 696539 | 4.98E+09 |

**Supplementary Table 1**. Overall and sex stratified AMI-associated mortality among United States adults with hypertension from 2000 to 2023.

| **Year** | **Medical Facility** | **Nursing home/Long Term Care** | **Hospices** | **Home** | **Other** |
| --- | --- | --- | --- | --- | --- |
| **2000** | 20618 | 5107 | Missing | 8881 | 837 |
| **2001** | 20290 | 5173 | Missing | 8915 | 910 |
| **2002** | 20365 | 5367 | Missing | 9573 | 1009 |
| **2003** | 20036 | 5472 | 11 | 9834 | 992 |
| **2004** | 19321 | 5191 | 20 | 9950 | 1101 |
| **2005** | 19681 | 5278 | 117 | 10185 | 1065 |
| **2006** | 18739 | 5136 | 146 | 10731 | 995 |
| **2007** | 17979 | 5099 | 229 | 10565 | 1089 |
| **2008** | 18511 | 5062 | 257 | 11064 | 1118 |
| **2009** | 17260 | 4564 | 269 | 10845 | 1141 |
| **2010** | 17392 | 4432 | 337 | 11544 | 1164 |
| **2011** | 17491 | 4543 | 350 | 11771 | 1388 |
| **2012** | 17302 | 4720 | 409 | 12228 | 1404 |
| **2013** | 17196 | 4713 | 458 | 12828 | 1416 |
| **2014** | 17184 | 4558 | 557 | 13406 | 1468 |
| **2015** | 17018 | 4674 | 593 | 14193 | 1596 |
| **2016** | 17150 | 4511 | 677 | 14547 | 1667 |
| **2017** | 17386 | 4564 | 758 | 15103 | 1708 |
| **2018** | 17563 | 4640 | 776 | 16173 | 1865 |
| **2019** | 17642 | 4549 | 926 | 16794 | 1964 |
| **2020** | 19845 | 4790 | 961 | 21719 | 2396 |
| **2021** | 20708 | 4361 | 1010 | 22774 | 2603 |
| **2022** | 19352 | 4409 | 1102 | 22056 | 2686 |
| **2023** | 17733 | 4150 | 1101 | 19790 | 2275 |
| **Total** | 443762 | 115063 | 11064 | 325469 | 35857 |

**Supplementary Table 2.** AMI-associated mortality among United States adults with hypertension stratified by place of death from 2000 to 2023.

**Age-Adjusted Rate (95% CI)**

| Year | Age 25-44 | Age 45-64 | Age 65+ |
| --- | --- | --- | --- |
| 2000 | 0.75 (0.70–0.81) | 10.39 (10.14–10.64) | 81.46 (80.51–82.40) |
| 2001 | 0.75 (0.69–0.80) | 10.10 (9.85–10.35) | 79.90 (78.96–80.83) |
| 2002 | 0.75 (0.69–0.80) | 10.12 (9.88–10.36) | 81.17 (80.24–82.11) |
| 2003 | 0.85 (0.79–0.92) | 10.22 (9.98–10.45) | 79.24 (78.32–80.16) |
| 2004 | 0.80 (0.74–0.86) | 10.06 (9.83–10.30) | 75.94 (75.05–76.84) |
| 2005 | 0.85 (0.79–0.92) | 10.06 (9.83–10.29) | 75.78 (74.89–76.66) |
| 2006 | 0.85 (0.79–0.92) | 10.01 (9.78–10.23) | 72.49 (71.63–73.34) |
| 2007 | 0.80 (0.74–0.86) | 9.51 (9.30–9.72) | 69.49 (68.66–70.32) |
| 2008 | 0.85 (0.79–0.92) | 9.95 (9.73–10.17) | 70.01 (69.19–70.84) |
| 2009 | 0.85 (0.79–0.92) | 9.49 (9.28–9.70) | 65.02 (64.23–65.81) |
| 2010 | 0.80 (0.74–0.86) | 9.53 (9.32–9.74) | 63.91 (63.14–64.69) |
| 2011 | 0.85 (0.79–0.92) | 9.75 (9.54–9.96) | 62.54 (61.78–63.30) |
| 2012 | 0.85 (0.79–0.92) | 9.77 (9.56–9.98) | 61.77 (61.02–62.51) |
| 2013 | 0.91 (0.84–0.98) | 9.65 (9.45–9.86) | 61.27 (60.53–62.00) |
| 2014 | 0.91 (0.84–0.98) | 9.93 (9.72–10.14) | 60.05 (59.34–60.77) |
| 2015 | 0.91 (0.84–0.98) | 10.03 (9.82–10.23) | 59.97 (59.26–60.68) |
| 2016 | 0.91 (0.84–0.98) | 10.31 (10.10–10.52) | 58.74 (58.05–59.43) |
| 2017 | 0.96 (0.89–1.03) | 10.31 (10.10–10.52) | 58.88 (58.20–59.56) |
| 2018 | 0.96 (0.89–1.03) | 10.61 (10.39–10.82) | 59.66 (58.98–60.33) |
| 2019 | 0.96 (0.90–1.03) | 10.33 (10.11–10.54) | 60.27 (59.60–60.94) |
| 2020 | 1.23 (1.15–1.30) | 12.63 (12.40–12.87) | 69.25 (68.54–69.96) |
| 2021 | 1.28 (1.20–1.36) | 13.15 (12.91–13.39) | 73.41 (72.67–74.15) |
| 2022 | 1.17 (1.10–1.25) | 12.13 (11.90–12.36) | 68.20 (67.50–68.89) |
| 2023 | 1.02 (0.95–1.09) | 10.88 (10.66–11.10) | 61.69 (61.03–62.35) |
| Total | 0.85 (0.84–0.87) | 10.13 (10.08–10.17) | 67.16 (66.99–67.33) |

**Supplementary Table 3.** Hypertension and AMI-associated AAMR per 100,000 stratified by Age Group in the United States from 2000-2023

**Age-Adjusted Rate (95% CI)**

| Year | Overall | Female | Male |
| --- | --- | --- | --- |
| 2000 | 19.84 (19.63 - 20.04) | 17.17 (16.92 - 17.42) | 22.91 (22.56 - 23.27) |
| 2001 | 19.43 (19.22 - 19.63) | 16.61 (16.37 - 16.85) | 22.64 (22.29 - 22.99) |
| 2002 | 19.68 (19.48 - 19.89) | 16.86 (16.62 - 17.10) | 22.92 (22.57 - 23.27) |
| 2003 | 19.39 (19.19 - 19.59) | 16.33 (16.09 - 16.57) | 22.90 (22.56 - 23.24) |
| 2004 | 18.67 (18.47 - 18.86) | 15.54 (15.31 - 15.77) | 22.40 (22.07 - 22.74) |
| 2005 | 18.66 (18.47 - 18.85) | 15.63 (15.40 - 15.85) | 22.14 (21.82 - 22.47) |
| 2006 | 18.00 (17.81 - 18.18) | 14.71 (14.49 - 14.93) | 21.91 (21.59 - 22.23) |
| 2007 | 17.22 (17.04 - 17.40) | 14.16 (13.95 - 14.37) | 20.76 (20.45 - 21.07) |
| 2008 | 17.49 (17.31 - 17.68) | 14.20 (13.99 - 14.41) | 21.38 (21.07 - 21.69) |
| 2009 | 16.36 (16.19 - 16.54) | 13.10 (12.90 - 13.30) | 20.15 (19.86 - 20.45) |
| 2010 | 16.13 (15.96 - 16.30) | 12.78 (12.58 - 12.97) | 20.17 (19.87 - 20.46) |
| 2011 | 15.97 (15.80 - 16.13) | 12.55 (12.35 - 12.74) | 20.02 (19.73 - 20.31) |
| 2012 | 15.82 (15.66 - 15.99) | 12.31 (12.12 - 12.50) | 20.01 (19.72 - 20.29) |
| 2013 | 15.71 (15.55 - 15.87) | 12.11 (11.92 - 12.30) | 19.96 (19.68 - 20.24) |
| 2014 | 15.57 (15.41 - 15.73) | 11.72 (11.54 - 11.90) | 20.13 (19.85 - 20.41) |
| 2015 | 15.58 (15.42 - 15.74) | 11.82 (11.64 - 12.00) | 19.97 (19.70 - 20.24) |
| 2016 | 15.44 (15.28 - 15.60) | 11.61 (11.43 - 11.79) | 19.92 (19.65 - 20.19) |
| 2017 | 15.49 (15.34 - 15.65) | 11.45 (11.27 - 11.62) | 20.23 (19.96 - 20.49) |
| 2018 | 15.75 (15.59 - 15.90) | 11.41 (11.24 - 11.59) | 20.85 (20.58 - 21.12) |
| 2019 | 15.77 (15.62 - 15.92) | 11.40 (11.23 - 11.57) | 20.91 (20.64 - 21.18) |
| 2020 | 18.44 (18.27 - 18.60) | 13.35 (13.16 - 13.53) | 24.44 (24.16 - 24.73) |
| 2021 | 19.45 (19.28 - 19.62) | 14.11 (13.92 - 14.30) | 25.67 (25.37 - 25.96) |
| 2022 | 18.04 (17.87 - 18.20) | 12.95 (12.77 - 13.14) | 24.03 (23.75 - 24.31) |
| 2023 | 16.26 (16.11 - 16.42) | 11.77 (11.60 - 11.94) | 21.47 (21.21 - 21.73) |
| Total | 17.10 (16.98 - 17.23) | 12.64 (12.51 - 12.77) | 21.97 (21.78 - 22.17) |
|  |  |  |  |

**Supplementary Table 4.** Overall and sex-stratified hypertension and AMI-associated AAMR per 100,000 in the United States from 2000-2023.

**Age-Adjusted Rate (95% CI)**

| **Year** | **NH American Indian** | **NH Black** | **NH White** | **Hispanic or Latino** |
| --- | --- | --- | --- | --- |
| **2000** | 16.18 (13.19 - 19.17) | 39.32 (38.30 - 40.33) | 18.11 (17.90 - 18.33) | 17.06 (16.17 - 17.96) |
| **2001** | 13.82 (11.13 - 16.52) | 37.79 (36.80 - 38.77) | 17.78 (17.57 - 17.99) | 17.56 (16.67 - 18.45) |
| **2002** | 18.13 (15.06 - 21.20) | 37.96 (36.98 - 38.94) | 17.99 (17.78 - 18.21) | 18.15 (17.27 - 19.03) |
| **2003** | 22.36 (18.97 - 25.76) | 36.61 (35.65 - 37.56) | 17.83 (17.62 - 18.04) | 17.62 (16.77 - 18.47) |
| **2004** | 16.84 (13.94 - 19.75) | 34.78 (33.86 - 35.70) | 17.30 (17.10 - 17.51) | 16.73 (15.93 - 17.54) |
| **2005** | 16.44 (13.68 - 19.21) | 33.54 (32.65 - 34.44) | 17.32 (17.12 - 17.53) | 17.82 (17.02 - 18.63) |
| **2006** | 18.48 (15.59 - 21.37) | 33.06 (32.18 - 33.93) | 16.68 (16.48 - 16.88) | 16.54 (15.78 - 17.29) |
| **2007** | 15.61 (12.97 - 18.26) | 30.45 (29.62 - 31.29) | 16.08 (15.89 - 16.28) | 15.39 (14.67 - 16.10) |
| **2008** | 16.86 (14.17 - 19.56) | 30.08 (29.26 - 30.90) | 16.48 (16.29 - 16.68) | 15.64 (14.94 - 16.34) |
| **2009** | 16.34 (13.72 - 18.97) | 27.80 (27.02 - 28.57) | 15.50 (15.31 - 15.68) | 14.17 (13.53 - 14.81) |
| **2010** | 17.25 (14.62 - 19.88) | 26.22 (25.48 - 26.97) | 15.29 (15.11 - 15.48) | 14.65 (14.01 - 15.29) |
| **2011** | 18.04 (15.49 - 20.59) | 26.00 (25.28 - 26.73) | 15.23 (15.04 - 15.41) | 14.07 (13.47 - 14.67) |
| **2012** | 16.85 (14.45 - 19.25) | 25.30 (24.59 - 26.00) | 15.14 (14.96 - 15.33) | 13.92 (13.34 - 14.50) |
| **2013** | 15.64 (13.39 - 17.88) | 25.54 (24.84 - 26.24) | 14.97 (14.79 - 15.15) | 14.41 (13.84 - 14.99) |
| **2014** | 17.81 (15.45 - 20.18) | 23.88 (23.22 - 24.54) | 15.13 (14.94 - 15.31) | 13.22 (12.69 - 13.75) |
| **2015** | 16.57 (14.42 - 18.72) | 23.42 (22.78 - 24.07) | 15.12 (14.94 - 15.30) | 13.54 (13.02 - 14.06) |
| **2016** | 15.88 (13.80 - 17.97) | 24.01 (23.37 - 24.65) | 14.98 (14.80 - 15.15) | 13.47 (12.96 - 13.97) |
| **2017** | 16.09 (13.99 - 18.20) | 23.12 (22.50 - 23.74) | 15.15 (14.97 - 15.33) | 13.32 (12.83 - 13.81) |
| **2018** | 17.37 (15.30 - 19.44) | 23.47 (22.86 - 24.08) | 15.49 (15.31 - 15.67) | 13.01 (12.54 - 13.49) |
| **2019** | 17.60 (15.50 - 19.70) | 23.23 (22.63 - 23.83) | 15.49 (15.31 - 15.66) | 13.62 (13.15 - 14.10) |
| **2020** | 19.47 (17.33 - 21.61) | 29.10 (28.44 - 29.77) | 17.59 (17.40 - 17.78) | 18.17 (17.63 - 18.71) |
| **2021** | 18.98 (16.83 - 21.12) | 29.38 (28.71 - 30.06) | 19.09 (18.89 - 19.28) | 17.31 (16.79 - 17.83) |
| **2022** | 17.56 (15.50 - 19.62) | 25.94 (25.32 - 26.56) | 18.14 (17.95 - 18.33) | 14.93 (14.46 - 15.41) |
| **2023** | 16.38 (14.43 - 18.33) | 23.50 (22.91 - 24.09) | 16.32 (16.14 - 16.50) | 13.58 (13.14 - 14.03) |

**Supplementary Table 5.** Hypertension and AMI-associated AAMR per 100,000 stratified by Race in the United States from 2000-2023

| **Place of Death** | **Deaths** | **% of Total Deaths** |
| --- | --- | --- |
| **Medical Facility** | 443762 | 47.56% |
| **Decedent's home** | 325469 | 34.88% |
| **Hospice facility** | 11064 | 1.19% |
| **Nursing home/long-term care** | 115063 | 12.33% |
| **Other** | 35857 | 3.84% |
| **Place of death unknown** | 1809 | 0.19% |
| **Total** | 933024 | 100% |

**Supplementary table 6.** Hypertension and AMI-associated mortality per 100,000 stratified by place of death in the United States from 2000 to 2023.

|  | Age Adjusted Rate (95% CI) |  |  |
| --- | --- | --- | --- |
| Year | **Metropolitan** | **Non-Metropolitan** |  |
| 2000 | 19.45 (19.22 - 19.67) | 21.71 (21.21 - 22.21) |  |
| 2001 | 19.03 (18.81 - 19.25) | 21.21 (20.72 - 21.70) |  |
| 2002 | 19.05 (18.83 - 19.27) | 22.55 (22.05 - 23.06) |  |
| 2003 | 18.65 (18.43 - 18.86) | 22.81 (22.30 - 23.31) |  |
| 2004 | 17.92 (17.71 - 18.13) | 22.16 (21.66 - 22.65) |  |
| 2005 | 17.78 (17.57 - 17.98) | 22.83 (22.33 - 23.33) |  |
| 2006 | 17.18 (16.98 - 17.38) | 21.95 (21.47 - 22.44) |  |
| 2007 | 16.32 (16.13 - 16.52) | 21.37 (20.89 - 21.85) |  |
| 2008 | 16.53 (16.34 - 16.72) | 22.04 (21.56 - 22.52) |  |
| 2009 | 15.28 (15.10 - 15.47) | 21.48 (21.01 - 21.95) |  |
| 2010 | 15.12 (14.94 - 15.31) | 21.02 (20.56 - 21.49) |  |
| 2011 | 14.91 (14.73 - 15.09) | 21.22 (20.75 - 21.68) |  |
| 2012 | 14.77 (14.59 - 14.94) | 21.16 (20.70 - 21.63) |  |
| 2013 | 14.67 (14.50 - 14.85) | 20.96 (20.50 - 21.42) |  |
| 2014 | 14.35 (14.18 - 14.52) | 21.73 (21.27 - 22.19) |  |
| 2015 | 14.24 (14.07 - 14.41) | 22.27 (21.80 - 22.73) |  |
| 2016 | 14.14 (13.97 - 14.30) | 22.26 (21.80 - 22.73) |  |
| 2017 | 14.12 (13.96 - 14.29) | 22.74 (22.27 - 23.21) |  |
| 2018 | 14.31 (14.15 - 14.47) | 23.46 (22.99 - 23.94) |  |
| 2019 | 14.23 (14.07 - 14.39) | 23.96 (23.48 - 24.44) |  |
| 2020 | 16.48 (16.31 - 16.65) | 28.96 (28.43 - 29.48) |  |

**Supplementary Table 7.** Hypertension and AMI-associated AAMR per 100,000 stratified by urban-rural classification in the United States from 2000 to 2020.

| State | Age-Adjusted Rate with 95% CI |
| --- | --- |
|  |  |
| Alabama | 8.72 (8.54 - 8.90) |
| Alaska | 4.97 (4.49 - 5.46) |
| Arizona | 7.33 (7.18 - 7.47) |
| Arkansas | 27.55 (27.14 - 27.95) |
| California | 12.54 (12.46 - 12.62) |
| Colorado | 6.89 (6.72 - 7.06) |
| Connecticut | 5.81 (5.65 - 5.97) |
| Delaware | 10.56 (10.11 - 11.00) |
| District of Columbia | 15.52 (14.81 - 16.24) |
| Florida | 7.86 (7.78 - 7.93) |
| Georgia | 9.03 (8.88 - 9.17) |
| Hawaii | 7.82 (7.51 - 8.12) |
| Idaho | 12.74 (12.35 - 13.14) |
| Illinois | 11.19 (11.07 - 11.32) |
| Indiana | 11.51 (11.33 - 11.68) |
| Iowa | 10.19 (9.96 - 10.41) |
| Kansas | 6.22 (6.03 - 6.41) |
| Kentucky | 15.08 (14.83 - 15.33) |
| Louisiana | 12.32 (12.10 - 12.55) |
| Maine | 8.46 (8.16 - 8.77) |
| Maryland | 13.20 (12.99 - 13.41) |
| Massachusetts | 6.87 (6.74 - 7.00) |
| Michigan | 9.97 (9.84 - 10.10) |
| Minnesota | 6.35 (6.21 - 6.50) |
| Mississippi | 24.86 (24.47 - 25.26) |
| Missouri | 11.98 (11.80 - 12.16) |
| Montana | 5.65 (5.35 - 5.95) |
| Nebraska | 6.24 (6.00 - 6.48) |
| Nevada | 5.18 (4.98 - 5.38) |
| New Hampshire | 7.39 (7.08 - 7.70) |
| New Jersey | 10.83 (10.68 - 10.97) |
| New Mexico | 7.70 (7.44 - 7.96) |
| New York | 9.71 (9.62 - 9.80) |
| North Carolina | 11.42 (11.28 - 11.57) |
| North Dakota | 11.61 (11.09 - 12.13) |
| Ohio | 14.97 (14.82 - 15.11) |
| Oklahoma | 11.20 (10.97 - 11.43) |
| Oregon | 8.38 (8.19 - 8.57) |
| Pennsylvania | 10.60 (10.48 - 10.71) |
| Rhode Island | 16.81 (16.31 - 17.30) |
| South Carolina | 14.13 (13.90 - 14.37) |
| South Dakota | 15.24 (14.70 - 15.79) |
| Tennessee | 15.89 (15.68 - 16.10) |
| Texas | 13.27 (13.16 - 13.38) |
| Utah | 5.50 (5.27 - 5.73) |
| Vermont | 11.83 (11.28 - 12.38) |
| Virginia | 8.39 (8.25 - 8.53) |
| Washington | 9.90 (9.74 - 10.07) |
| West Virginia | 14.18 (13.84 - 14.52) |
| Wisconsin | 12.04 (11.85 - 12.22) |
| Wyoming | 11.64 (11.01 - 12.26) |

**Supplementary Table 8.** Hypertension and AMI-associated AAMR per 100,000 stratified by state in the United States from 2000-2019

| Census Region | Year | Age-Adjusted Rate with 95% CI |
| --- | --- | --- |
| Census Region 1: Northeast | 2000 | 18.44 (18.01 - 18.87) |
| Census Region 1: Northeast | 2001 | 18.39 (17.96 - 18.82) |
| Census Region 1: Northeast | 2002 | 18.53 (18.10 - 18.96) |
| Census Region 1: Northeast | 2003 | 17.49 (17.07 - 17.90) |
| Census Region 1: Northeast | 2004 | 17.16 (16.75 - 17.57) |
| Census Region 1: Northeast | 2005 | 16.52 (16.13 - 16.92) |
| Census Region 1: Northeast | 2006 | 15.79 (15.41 - 16.18) |
| Census Region 1: Northeast | 2007 | 15.13 (14.75 - 15.50) |
| Census Region 1: Northeast | 2008 | 15.33 (14.95 - 15.71) |
| Census Region 1: Northeast | 2009 | 14.04 (13.68 - 14.40) |
| Census Region 1: Northeast | 2010 | 13.92 (13.56 - 14.27) |
| Census Region 1: Northeast | 2011 | 13.89 (13.53 - 14.24) |
| Census Region 1: Northeast | 2012 | 13.96 (13.61 - 14.31) |
| Census Region 1: Northeast | 2013 | 13.59 (13.24 - 13.93) |
| Census Region 1: Northeast | 2014 | 13.67 (13.32 - 14.01) |
| Census Region 1: Northeast | 2015 | 13.32 (12.98 - 13.66) |
| Census Region 1: Northeast | 2016 | 12.80 (12.47 - 13.12) |
| Census Region 1: Northeast | 2017 | 12.44 (12.12 - 12.76) |
| Census Region 1: Northeast | 2018 | 12.52 (12.20 - 12.84) |
| Census Region 1: Northeast | 2019 | 12.36 (12.04 - 12.67) |
| Census Region 1: Northeast | 2020 | 14.73 (14.39 - 15.08) |
| Census Region 1: Northeast | 2021 | 14.06 (13.72 - 14.39) |
| Census Region 1: Northeast | 2022 | 13.25 (12.93 - 13.57) |
| Census Region 1: Northeast | 2023 | 11.92 (11.61 - 12.22) |
| Census Region 2: Midwest | 2000 | 21.09 (20.66 - 21.53) |
| Census Region 2: Midwest | 2001 | 20.17 (19.75 - 20.60) |
| Census Region 2: Midwest | 2002 | 19.96 (19.54 - 20.38) |
| Census Region 2: Midwest | 2003 | 19.50 (19.09 - 19.91) |
| Census Region 2: Midwest | 2004 | 18.50 (18.10 - 18.89) |
| Census Region 2: Midwest | 2005 | 18.88 (18.48 - 19.28) |
| Census Region 2: Midwest | 2006 | 18.31 (17.91 - 18.70) |
| Census Region 2: Midwest | 2007 | 17.45 (17.07 - 17.83) |
| Census Region 2: Midwest | 2008 | 17.77 (17.39 - 18.15) |
| Census Region 2: Midwest | 2009 | 16.69 (16.32 - 17.05) |
| Census Region 2: Midwest | 2010 | 16.12 (15.76 - 16.48) |
| Census Region 2: Midwest | 2011 | 16.60 (16.24 - 16.96) |
| Census Region 2: Midwest | 2012 | 16.17 (15.81 - 16.52) |
| Census Region 2: Midwest | 2013 | 16.03 (15.68 - 16.38) |
| Census Region 2: Midwest | 2014 | 16.00 (15.65 - 16.34) |
| Census Region 2: Midwest | 2015 | 15.81 (15.47 - 16.15) |
| Census Region 2: Midwest | 2016 | 15.32 (14.99 - 15.65) |
| Census Region 2: Midwest | 2017 | 15.50 (15.16 - 15.83) |
| Census Region 2: Midwest | 2018 | 15.86 (15.52 - 16.19) |
| Census Region 2: Midwest | 2019 | 15.39 (15.07 - 15.72) |
| Census Region 2: Midwest | 2020 | 17.29 (16.94 - 17.63) |
| Census Region 2: Midwest | 2021 | 18.32 (17.96 - 18.68) |
| Census Region 2: Midwest | 2022 | 17.16 (16.81 - 17.50) |
| Census Region 2: Midwest | 2023 | 15.37 (15.04 - 15.69) |
| Census Region 3: South | 2000 | 20.96 (20.60 - 21.31) |
| Census Region 3: South | 2001 | 20.49 (20.14 - 20.84) |
| Census Region 3: South | 2002 | 20.85 (20.50 - 21.20) |
| Census Region 3: South | 2003 | 20.97 (20.63 - 21.32) |
| Census Region 3: South | 2004 | 19.99 (19.65 - 20.33) |
| Census Region 3: South | 2005 | 20.39 (20.05 - 20.72) |
| Census Region 3: South | 2006 | 19.26 (18.93 - 19.58) |
| Census Region 3: South | 2007 | 18.58 (18.27 - 18.90) |
| Census Region 3: South | 2008 | 18.70 (18.39 - 19.01) |
| Census Region 3: South | 2009 | 17.97 (17.67 - 18.28) |
| Census Region 3: South | 2010 | 17.61 (17.31 - 17.90) |
| Census Region 3: South | 2011 | 17.07 (16.78 - 17.36) |
| Census Region 3: South | 2012 | 17.07 (16.79 - 17.35) |
| Census Region 3: South | 2013 | 17.10 (16.82 - 17.38) |
| Census Region 3: South | 2014 | 17.05 (16.77 - 17.32) |
| Census Region 3: South | 2015 | 17.08 (16.81 - 17.36) |
| Census Region 3: South | 2016 | 17.24 (16.97 - 17.51) |
| Census Region 3: South | 2017 | 17.55 (17.28 - 17.82) |
| Census Region 3: South | 2018 | 18.17 (17.90 - 18.44) |
| Census Region 3: South | 2019 | 18.51 (18.24 - 18.78) |
| Census Region 3: South | 2020 | 22.14 (21.85 - 22.44) |
| Census Region 3: South | 2021 | 23.93 (23.62 - 24.24) |
| Census Region 3: South | 2022 | 21.93 (21.64 - 22.22) |
| Census Region 3: South | 2023 | 19.97 (19.70 - 20.25) |
| Census Region 4: West | 2000 | 17.87 (17.43 - 18.31) |
| Census Region 4: West | 2001 | 17.75 (17.32 - 18.19) |
| Census Region 4: West | 2002 | 18.53 (18.09 - 18.96) |
| Census Region 4: West | 2003 | 18.40 (17.97 - 18.83) |
| Census Region 4: West | 2004 | 18.09 (17.67 - 18.51) |
| Census Region 4: West | 2005 | 17.56 (17.15 - 17.97) |
| Census Region 4: West | 2006 | 17.46 (17.06 - 17.86) |
| Census Region 4: West | 2007 | 16.50 (16.11 - 16.88) |
| Census Region 4: West | 2008 | 17.19 (16.80 - 17.58) |
| Census Region 4: West | 2009 | 15.37 (15.01 - 15.74) |
| Census Region 4: West | 2010 | 15.64 (15.28 - 16.01) |
| Census Region 4: West | 2011 | 15.25 (14.90 - 15.60) |
| Census Region 4: West | 2012 | 14.99 (14.64 - 15.33) |
| Census Region 4: West | 2013 | 14.97 (14.63 - 15.31) |
| Census Region 4: West | 2014 | 14.24 (13.92 - 14.57) |
| Census Region 4: West | 2015 | 14.62 (14.29 - 14.95) |
| Census Region 4: West | 2016 | 14.69 (14.37 - 15.02) |
| Census Region 4: West | 2017 | 14.51 (14.19 - 14.83) |
| Census Region 4: West | 2018 | 14.28 (13.97 - 14.59) |
| Census Region 4: West | 2019 | 14.32 (14.01 - 14.63) |
| Census Region 4: West | 2020 | 16.23 (15.91 - 16.56) |
| Census Region 4: West | 2021 | 17.40 (17.06 - 17.74) |
| Census Region 4: West | 2022 | 16.18 (15.86 - 16.50) |
| Census Region 4: West | 2023 | 14.29 (13.99 - 14.59) |

**Supplementary Table 9.** Hypertension and AMI-associated AAMR per 100,000 stratified by census region in the United States from 2000-2023.

| **UCD - 15 Leading Causes of Death** | **Deaths** |
| --- | --- |
| **Diseases of heart (I00-I09,I11,I13,I20-I51)** | 705869 |
| **Diabetes mellitus (E10-E14)** | 94958 |
| **Chronic lower respiratory diseases (J40-J47)** | 14172 |

**Supplementary table 10**. Hypertension and AMI-associated deaths per 100,000 by top 3 underlying causes of death in the United States from 2000-2023.

| **Interval of years** | | | **APC (95% CI)** | |
| --- | --- | --- | --- | --- |
| **Overall** |  | |  |  |
| 2000–2012 | -2.09 (-3.42 to -1.76) | |  |  |
| 2012–2018 | -0.39 (-1.56 to 1.31) | |  |  |
| 2018–2021 | 8.38 (6.22 to 10.00) | |  |  |
| 2021–2023 | -8.59 (-11.44 to -6.04) | |  |  |
| **Male** |  | |  |  |
| 2000–2011 | -1.44 (-3.09 to -1.02) | |  |  |
| 2011–2018 | 0.15 (-0.83 to 1.71) | |  |  |
| 2018–2021 | 8.67 (6.50 to 10.23) | |  |  |
| 2021–2023 | -8.63 (-11.39 to -6.11) | |  |  |
| **Female** |  | |  |  |
| 2000–2012 | -2.95 (-4.51 to -2.61) | |  |  |
| 2012–2018 | -1.52 (-2.65 to 0.15) | |  |  |
| 2018–2021 | 8.03 (5.59 to 9.77) | |  |  |
| 2021–2023 | -8.54 (-11.68 to -5.70) | |  |  |
| **American Indian or Alaska Native** | | |  |  |
| 2000–2023 | 0.13 (-0.44 to -0.82) | |  |  |
| **Black or African American** | | |  |  |
| 2000–2012 | -3.85 (-6.21 to -3.31) | |  |  |
| 2012–2018 | -1.61 (-3.48 to 0.83) | |  |  |
| 2018–2021 | 9.33 (5.78 to 11.93) | |  |  |
| 2021–2023 | -11.70 (-15.95 to -7.58) | |  |  |
| **White** |  | |  |  |
| 2000–2011 | -1.68 (-2.78 to -1.36) | |  |  |
| 2011–2018 | 0.03 (-1.16 to 1.64) | |  |  |
| 2018–2021 | 8.10 (5.98 to 9.63) | |  |  |
| 2021–2023 | -6.85 (-9.61 to -4.35) | |  |  |
| **Hispanic or Latino** |  | |  |  |
| 2000-2018 | -1.88 (-2.50 to -1.39) | |  |  |
| 2018-2021 | 12.39 (7.24 to 16.02) | |  |  |
| 2021-2023 | -14.60 (-20.06 to -8.34) | |  |  |
| **Northeast** | | |  |  |
| 2000–2009 | -3.03 (-5.92 to -2.28) | |  |  |
| 2009–2018 | -1.66 (-2.70 to -0.09) | |  |  |
| 2018–2021 | 5.17 (2.14 to 7.20) | |  |  |
| 2021–2023 | -9.11 (-13.08 to -4.58) | |  |  |
| **Midwest** |  | |  |  |
| 2000–2010 | -2.34 (-4.00 to -1.91) | |  |  |
| 2010–2018 | -0.92 (-1.73 to 0.49) | |  |  |
| 2018–2021 | 6.02 (3.62 to 7.53) | |  |  |
| 2021–2023 | -7.75 (-10.96 to -4.62) | |  |  |
| **South** |  | |  |  |
| 2000–2003 | 0.02 (-1.65 to 3.54) | |  |  |
| 2003–2012 | -2.32 (-4.48 to -1.87) | |  |  |
| 2012–2018 | 0.75 (-0.60 to 2.41) | |  |  |
| 2018–2021 | 10.76 (8.46 to 12.40) | |  |  |
| 2021–2023 | -8.59 (-11.49 to -5.56) | |  |  |
| **West** |  | |  |  |
| 2000–2018 | -1.64 (-2.08 to -1.29) | |  |  |
| 2018–2021 | 7.80 (3.08 to 9.97) | |  |  |
| 2021–2023 | -8.89 (-14.45 to -2.09) | |  |  |
| **Metropolitan** | | |  |  |
| 2000–2011 | -2.59 (-3.98 to -2.18) | |  |  |
| 2011–2018 | -0.95 (-2.00 to 0.62) | |  |  |
| 2018–2020 | 7.53 (3.20 to 10.05) | |  |  |
| **Non-Metropolitan** | | |  |  |
| 2000–2013 | -0.38 (-2.32 to 1.69) | |  |  |
| 2013–2018 | 1.58 (-0.43 to 3.42) | |  |  |
| 2018–2020 | 10.81 (5.58 to 14.10) | |  |  |
| **Young Adults** | | |  |  |
| 2000–2018 | 1.11 (0.50 to 1.54) | |  |  |
| 2018–2021 | 11.48 (6.82 to 14.26) | |  |  |
| 2021–2023 | -10.91 (-17.55 to -3.56) | |  |  |
| **Middle Aged Adults** | | |  |  |
| 2000–2010 | -0.79 (-3.69 to -0.001) | |  |  |
| 2010–2018 | 0.97 (-0.30 to 2.93) | |  |  |
| 2018–2021 | 8.56 (5.64 to 10.52) | |  |  |
| 2021–2023 | -9.09 (-13.21 to -5.08) | |  |  |
| **Elderly Adults** | | |  |  |
| 2000–2003 | -0.76 (-2.19 to 2.17) | |  |  |
| 2003–2011 | -3.01 (-4.90 to -2.54) | |  |  |
| 2011–2018 | -0.97 (-1.74 to 0.33) | |  |  |
| 2018–2021 | 8.23 (6.50 to 9.49) | |  |  |
| 2021-2023 -8.27 (-10.58 to -6.28) | | |  |  |

**Supplementary Table 11**. Summary APCs of Hypertension and AMI-associated AAMR per 100,000 in the United States from 2000-2023.
